# The first report on effect of fecal microbiota transplantation as a complementary treatment in a patient with steroid-refractory Cronkhite-Canada syndrome: A case report

**DOI:** 10.1097/MD.0000000000029135

**Published:** 2022-03-25

**Authors:** Sun Young Kim, Jongbeom Shin, Jin-Seok Park, Boram Cha, Youjeong Seo, Soo-Hyun Park, Jung Hwan Lee, Jun-Seob Kim, Gyesook Kwon

**Affiliations:** a *Digestive Disease Center, Department of Internal Medicine, Inha University College of Medicine, Incheon, South Korea,*; b *Department of Pathology, Inha University College of Medicine, Incheon, South Korea,*; c *Department of Hospital Medicine, Inha University College of Medicine, Incheon, South Korea,*; d *Department of Nano-Bioengineering, Incheon National University, Incheon, South Korea.*

**Keywords:** case report, Cronkhite-Canada syndrome, fecal microbiota transplantation, gut microbiome

## Abstract

**Rationale:**

Cronkhite-Canada syndrome (CCS) is a rare non-hereditary disease of unknown etiology that is characterized by the appearance of multiple polyps in the entire gastrointestinal (GI) tract, except in the esophagus, with GI and non-GI symptoms. Various factors are associated with the pathogenesis of CCS. Immune dysregulation has been discussed as one of the pathogeneses of CCS, and dysbiosis of the gut microbiota can affect the immune system. Currently, standard treatment has not been established.

**Patient concerns and diagnosis:**

We present the treatment with fecal microbiota transplantation (FMT) in a 67-year-old male patient with steroid-refractory CCS who could not undergo anti-tumor necrosis factor-a treatment due to suspected tuberculosis infection.

**Interventions:**

FMT has recently attracted attention as a method of overcoming drug resistance through immunomodulatory effects through microbiome regulation. We collected the patient's stool samples before FMT and 8weeks after FMT.

**Outcomes:**

We analyzed the microbiome composition of patients by sequencing the V3-V4 region of the 16s rRNA gene (Miseq). After FMT, the number of episodes of diarrhea and hypoalbuminemia were also corrected. The Chao 1 index after FMT, which was significantly higher than that of donors before FMT, changed to a similar level for donors after FMT. *Fusobacterium nucleatum*, *Pyramidobacter piscolens*, and *Campylobacter concisus* disappeared after FMT, suggesting the presence of an association between gut microbiota and CCS.

**Lessons:**

Furthermore, we provide the possibility that microbiome modulation by FMT could serve as a complementary treatment in patients with steroid-refractory CCS.

## 1. Introduction

Cronkhite-Canada syndrome (CCS) is a rare non-hereditary disease of unknown etiology that was first described in 1955.^[1]^ CCS is characterized by the appearance of multiple polyps in the entire gastrointestinal (GI) tract, except the esophagus, and GI symptoms, such as diarrhea and digestive disorders, hair loss, nail atrophy, and hyperpigmentation of the skin. CCS is diagnosed on the basis of clinical findings, endoscopy, and pathological findings.^[2]^ Most patients are diagnosed at over 50years of age, with a male-to-female ratio of 3:2 and a slightly higher incidence in men. Malnutrition, GI bleeding, and infection are common, with a mortality rate of up to 60%; however, the prognosis is poor, and the pathogenesis remains unknown.^[3]^

The treatments include hyperalimentation, corticosteroids, H2-receptor antagonists, antibiotics, acid suppression, cromolyn sodium, anabolic steroids, surgery, and combinations of these therapies. However, the optimal medical therapy for CCS remains unclear. In general, steroid therapy can be considered first, but other treatments should be considered in cases of refractory to steroid therapy. Recently, an anti-TNF (tumor necrosis factor)-a agent was reported to improve when administered in steroid-refractory CCS.^[4]^

Fecal microbiota transplantation (FMT) is widely used as a treatment for *Clostridioides difficile* infection (CDI),^[5,6]^ and recently, research is being conducted for applications other than CDI. It is effective in patients with irritable bowel syndrome who complain of diarrhea and in patients with inflammatory bowel disease whose main etiology is chronic mucosal inflammation.^[6]^ There is also a report that the drug response was restored in patients with melanoma and colorectal cancer who were resistant to anti-PD-1 treatment.^[7]^

We report a case of CCS with a nephrotic syndrome that was refractory to steroid treatment and could not be treated with anti-TNF-a due to suspected active tuberculosis infection and improved by FMT.

## 2. Case report

**Figure 1.**
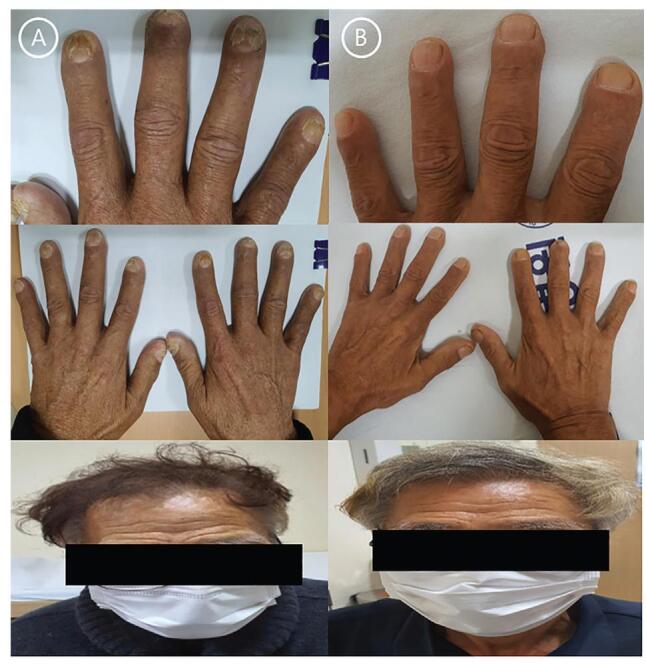
(A) The patient shows nail dystrophy, hyperpigmentation, and hair loss. (B) After FMT, these were improved. FMT = fecal microbiota transplantation.

**Figure 2.**
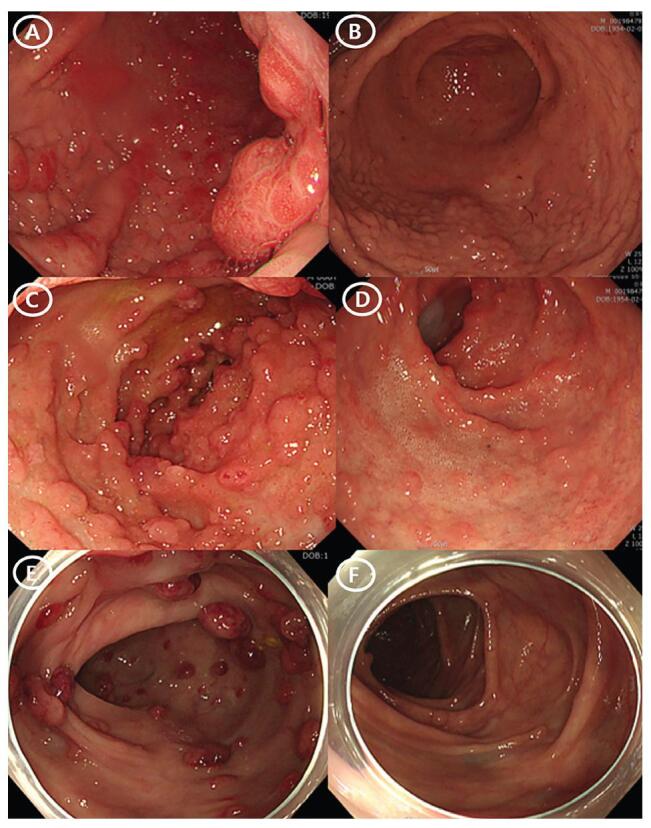
Compared with the endoscopy performed at the time of diagnosis, it was confirmed that the number and size of polyps in the (A,B) antrum and (C,D) duodenum were reduced after FMT. The number and size of (E,F) colon polyps were significantly reduced compared to the time of FMT. FMT = fecal microbiota transplantation.

**Figure 3.**
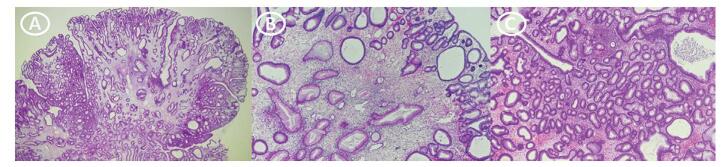
(A) Dilated mucosal gland with edematous stroma and mild inflammatory cells infiltration, consistent with (B) juvenile polyp, focally with (C) inflammatory cell infiltration. In the upper right half of the same picture, inflammatory cells, and among them neutrophils, are contained in the stretched gland, and crypt abscess is observed.

A 67-year-old man was admitted to our hospital for evaluation of nephrotic syndrome and chronic diarrhea due to edema of both legs that occurred 4 weeks before admission. The patient was taking medication for diabetes and had a history of tuberculosis. Diarrhea more than 5 times a day lasted for a month and continued without improvement. Upon admission, his blood pressure was 126/66mmHg, pulse rate was 69 beats per minute, respiratory rate was 18 beats per minute, and body temperature was 36.8°C. Figure 1A shows the patient's hair loss, hyperpigmentation, and nail dystrophy. Peripheral blood tests showed leukocyte count (18,450/mm^3^), hemoglobin level (8.9 g/dL), and platelet count (247,000/mm^3^). In biochemical tests, the level of total protein was 4.1 g/dL, albumin 1.6 g/dL, and blood urea nitrogen/creatinine 30.3/1.31 mL/dL. Na/K/Cl concentration was 134/3.22/104mEq/L, and other blood tests were unremarkable. Molecular genetic stool tests were performed for persistent diarrhea, but no pathogens were identified. The stool occult blood test result was positive, and protein-losing enteropathy was confirmed by the fecal alpha-1 antitrypsin test. Endoscopies of the stomach and strawberry-shaped polyps covered the entire GI tract, except for the esophagus, and multiple polyps were also observed in the duodenum. During a colonoscopy, numerous sessile or pedunculated polyps were observed in the entire large intestine, including the distal ileum (Fig. 2). A polypectomy for diagnosis was performed during the colonoscopy. Histological examination of the polyp revealed enlarged cystic glands and edematous stroma, without atypia (Fig. 3). The diagnosis of CCS was established by combining clinical and histological findings. The patient was treated with corticosteroids (prednisolone 20 mg orally daily) with rifaximin for 2 weeks. However, diarrhea did not improve, and general weakness worsened. He was hospitalized for intravenous steroid administration and nutrition through enteral and parenteral routes. After hospitalization, 40mg (1 mg/kg) of methylprednisolone was administered. Although high-dose steroids were administered intravenously, the patient complained of watery diarrhea 5 to 7 times per day, and his albumin level did not improve. According to the guidelines, the patient needed to consider treatment escalation, such as a TNF-a inhibitor. The patient had a prior history of tuberculosis, and a chest X-ray scan showed lesions in the both upper lobes, presenting active tuberculosis. Treatment with tuberculosis is necessary for the administration of anti-TNF-a, such as infliximab. However, the patient's diarrhea was persistent, and his general weakness worsened, making it difficult to obtain a treatment period for tuberculosis. Therefore, the use of FMT that can modify steroidrefractory conditions was discussed with the patient.

The FMT donor was a 38-year-old man who was not prescribed antibiotics and had no GI or other health problems. He underwent blood, stool, and psychological tests and answered specific questions according to the FMT guidelines. The patient underwent bowel preparation, and the fecal solution was delivered to the colon using colonoscopy.

After FMT, the number of diarrhea episodes in the patient decreased rapidly, and the patient's albumin level gradually improved. Steroids were reduced step-by-step and finally stopped after 4 months. Both clinical symptoms and endoscopic findings improved (Figs. 1 and 2). A year after FMT, the patient was asymptomatic and did not require medication upon outpatient follow-up. Additionally, a sputum test was performed, and there was no tuberculosis infection, and a chest computed tomography scan showed no change in the lesion.

## 3. Discussion

This case describes the improvement in a patient with steroidrefractory CCS after FMT. The etiology of CCS remains unclear. Because of this complex etiology, definitive standard treatment for CCS has not been established. Corticosteroids are recommended as the mainstay of medical treatment.^[8]^ An oral steroid preparation, prednisone 20 to 60 mg per day, was administered at an initial therapeutic dose, with the additional nutrition therapy as a supplementary therapy. In cases of non-response to steroid treatment, immunomodulatory treatments, such as anti-TNF-a and azathioprine treatment, can improve it, and the reduction of inflammation is attracting attention as a mechanism of treatment.^[9]^ However, for cases wherein anti-TNF-a treatment is not possible and the administration of slow active agents, such as azathioprine, is limited to use as a therapeutic agent, an appropriate treatment to replace it has not yet been established.

CCS is associated with immune mechanisms.^[4]^ The gut microbiota provides an intestinal biological barrier against pathogens and has a pivotal role in the maintenance of intestinal homeostasis and modulation of the host immune system.^[10]^ The specific changes in the composition of gut microbiota, termed dysbiosis, have been associated not only with many GI diseases but also with metabolic diseases, autoimmune diseases, cancer, cardiovascular and central nervous system disorders, and neuropsychiatric disorders.

FMT is the infusion of liquid filtrate feces from a healthy donor into the gut of a recipient to cure a specific disease, such as GI diseases, including CDI, metabolic diseases, autoimmune diseases, and allergic disorders. A clinical trial evaluated the safety and efficacy of responder-derived FMT together with anti-PD-1 in patients with PD-1-refractory melanoma to investigate whether resistance to anti-PD-1 can be overcome by changing the gut microbiota.^[11]^ Here, an increase in taxa was associated with responses to anti-PD-1, increased CD8+ T cell activation, and decreased frequency of interleukin-8 expressing myeloid cells. This result has distinct proteomic and metabolomic signatures and confirmed that the gut microbiome regulates these changes.^[11]^ The restoration of the microbiome ecosystem might improve the symptoms of steroid-refractory patients. Therefore, to improve the steroid-refractory state, stool from a healthy male was transplanted.

**Figure 4.**
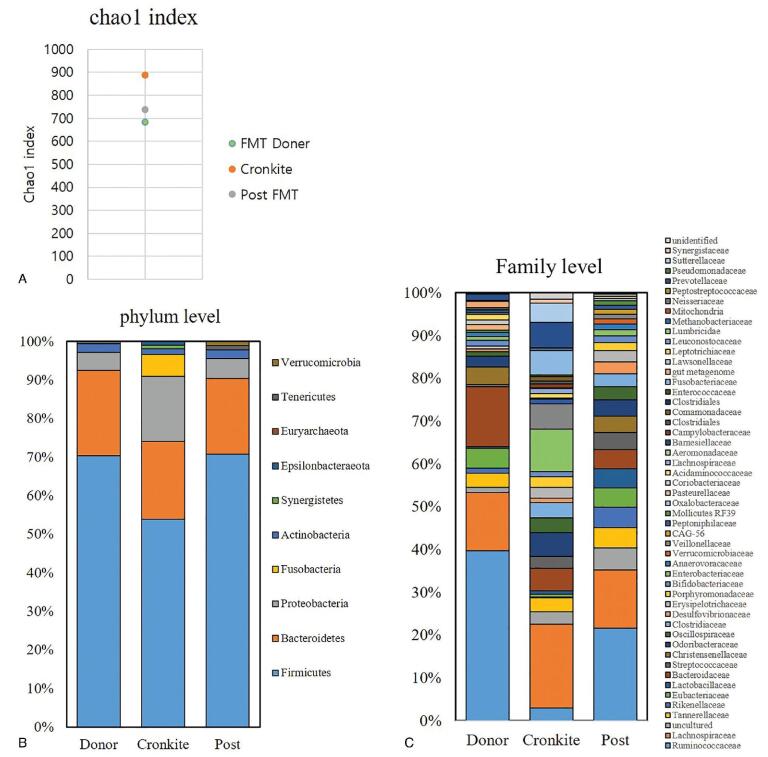
(A) In patients with CCS, the Chao 1 index for species richness was increased compared to donor and post-FMT. (B) At the phylum level, fusobacteria, synergistetes, and epsilonbacteraeota. (C) At the family level, *Fusobacterum nucleatum*, *Pyramidobacter piscolens,* and *Campylobacter concisus*, which were not observed in donors and after FMT, were observed in patients with CCS. CCS = Cronkhite-Canada syndrome, FMT = fecal microbiota transplantation.

We collected the patient's stool sample before FMT and 8 weeks after FMT, and the differences in microbiome composition of frozen stool samples before and after FMT treatment were analyzed. The microbiome composition was assessed by sequencing of the V3-V4 region of the 16s rRNA gene (Miseq). QIIME (2021.8) was used for the operational taxonomic unit analysis and taxonomy information. The major sequence of each operational taxonomic unit was referred to the NCBI 16S database, and taxonomy information was obtained using the Silva 132 database. To compare the relative abundance of microbial communities in the pre- and post-FMT, a bar graph was created for each genus, representing at least 1%. In the patient with CCS, the Chao 1 index was increased compared to that in the FMT donor, but decreased to a similar level after FMT. In a healthy state, the diversity of the microbiome is maintained without increasing or decreasing excessively.^[12]^ The excessive increase in alpha diversity seen in patients with CCS is presumed to be caused by an imbalance in the intestinal microbiota. At the family level, *Fusobacterum nucleatum*, *Pyramidobacter piscolens*, and *Campylobacter concisus*, which were not observed in donors, were observed in patients with CCS. *F neucleatum* is known to be abundantly found in colorectal cancer and inflammatory bowel disease patients.^[13]^*P piscolens* is related to periodontal disease and abscess.^[14]^*C concisus*, belonging to epsilonbacteraeota, is associated with active Crohn disease.^[15]^ After FMT, we found that these bacteria disappeared, and the composition of the microbiome was changed to be similar to that of a healthy donor (Fig. 4). Therefore, we suggest that FMT corrects the microbiome dysbiosis in patients with CCS, and that FMT alters the abundance of microbiota. The therapeutic effect of FMT may be related to changes in the gut microbiota. Further research is required to determine the specific mechanism and role of these bacteria.

In conclusion, although CCS is rare, it is a serious disease with increased mortality without appropriate treatment. The optimal treatment for steroid-refractory patients for whom immunomodulatory treatment is less effective or impossible has not been established. We provide the possibility that FMT could serve as a complementary treatment in patients with steroid-refractory CCS. This case is the first to report the improvement of CCS through microbiome modulation by FMT in steroid-refractory patients whose use of immunomodulators is restricted. This is also the first case report analyzing the microbiome of a patient with CCS. Further investigations on the therapeutic effect of FMT and the microbiome characteristics in patients with CCS are needed.

## Acknowledgments

The authors would like to thank the patient and his family. This case study was conducted according to the tenets of the declaration of Helsinki and approved by the institutional Review Board of Inha University Hospital (2021-10-030). Our patient provided informed written consent. The data that support the findings of this study are available from the corresponding author, [J Shin], upon reasonable request.

## Author contributions

Concept and design of the study, and acquisition, analysis, and interpretation of the data: Sun Young Kim and Jongbeom Shin. Collection and assembly of data: Jongbeom Shin, Boram Cha, Sun Young Kim, and Gyesook Kwon. Analysis and interpretation of the data: Youjeong Seo, Sun Young Kim, Jongbeom Shin, and Jun-seob Kim. Drafting of the article: Sun Young Kim and Jongbeom Shin. Critical revision of the manuscript: Jongbeom Shin.

**Conceptualization:** Sun Young Kim, Jongbeom Shin.

**Data curation:** Jin-Seok Park MD, Boram Cha, Soo-Hyun Park, Jung Hwan Lee.

**Formal analysis:** Youjeong Seo, Jun-Seob Kim, Gyesook Kwon.

**Writing** - **original draft:** Sun Young Kim.
